# Assessment of corporate compliance with guidance and regulations on labels of commercially produced complementary foods sold in Cambodia, Nepal, Senegal and Tanzania

**DOI:** 10.1111/mcn.12268

**Published:** 2016-04-15

**Authors:** Lara Sweet, Catherine Pereira, Rosalyn Ford, Alison B Feeley, Jane Badham, Khin Mengkheang, Indu Adhikary, Ndèye Yaga Sy Gueye, Aminata Ndiaye Coly, Cecilia Makafu, Elizabeth Zehner

**Affiliations:** ^1^ JB Consultancy Bryanston South Africa; ^2^ Helen Keller International Phnom Penh Cambodia; ^3^ Helen Keller International Kathmandu Valley Nepal; ^4^ Helen Keller International Dakar Senegal; ^5^ Helen Keller International Dar es Salaam Tanzania; ^6^ Helen Keller International Washington, D.C. USA

## Abstract

National legislation and global guidance address labelling of complementary foods to ensure that labels support optimal infant and young child feeding practices. This cross‐sectional study assessed the labels of commercially produced complementary foods (CPCF) sold in Phnom Penh (*n* = 70), Cambodia; Kathmandu Valley (*n* = 22), Nepal; Dakar Department (*n* = 84), Senegal; and Dar es Salaam (*n* = 26), Tanzania. Between 3.6% and 30% of products did not provide any age recommendation and 8.6−20.2% of products, from all sites, recommended an age of introduction of <6 months. Few CPCF products provided a daily ration (0.0−8.6%) and 14.5−55.6% of those that did exceeded the daily energy recommendation for complementary foods for a breastfed child from 6 to 8.9 months of age. Only 3.6−27.3% of labels provided accurate and complete messages in the required language encouraging exclusive breastfeeding, and almost none (0.0−2.9%) provided accurate and complete messages regarding the appropriate introduction of complementary foods together with continued breastfeeding. Between 34.3% and 70.2% of CPCF manufacturers also produced breastmilk substitutes and 41.7−78.0% of relevant CPCF products cross‐promoted their breastmilk substitutes products. Labelling practices of CPCF included in this study do not fully comply with international guidance on their promotion and selected aspects of national legislation, and there is a need for more detailed normative guidance on certain promotion practices in order to protect and promote optimal infant and young child feeding.

**Key messages box**

- Current labelling practices of commercially produced complementary foods sold in Phnom Penh, Cambodia; Kathmandu Valley, Nepal; Dakar Department, Senegal; and Dar es Salaam, Tanzania are not optimal and often do not follow national legislative requirements or best‐practice.
- Manufacturers of commercially produced complementary foods should ensure that products are appropriately labelled to support global infant and young child feeding messages and comply with national law and best‐practice
- Labelling regulations should be strengthened and enforced to prevent practices that undermine breastfeeding and optimal complementary feeding.
- Specific and detailed global guidance on what defines inappropriate promotion and the importance of preventing cross‐promotion with breastmilk substitutes is necessary to ensure products promote optimal infant and young child feeding.

## Introduction

Poor complementary feeding practices are a concern in many low and middle income countries where malnutrition is a public health concern. The State of the World's Children (2015) reported that 92% of Tanzanian, 88% of Cambodian, 67% of Senegalese and 66% of Nepali children are introduced to complementary foods at the age of 6–8 months (UNICEF 2015). However, some studies show that complementary foods or beverages other than breastmilk are often given to children before the recommended age of 6 months (Kimani‐Murage et al. 2011; Radwan 2013).

Commercially produced complementary foods (CPCF) are an option for families who can afford them and have the knowledge and facilities to prepare and feed them safely (WHO 2003). Nutritionally adequate, safe and affordable, fortified complementary foods and nutrient supplements can contribute to population health and may be needed in emergency situations as part of a broader strategy, including continued breastfeeding and the use of culturally appropriate, local family foods, to meet the nutritional needs of older infants and young children (UNICEF 2011; WHO 2003, PAHO/WHO 2003). Inappropriate marketing of CPCF could however undermine optimal breastfeeding practices (Lutter, 2003; Piwoz et al. 2003) by, for example, encouraging their early introduction or recommending an excessive daily ration that interferes with continued breastfeeding (Quinn et al. 2010).

Food labels provide basic product information to the users on health, safety and nutrition and serve as a vehicle for food marketing, promotion and advertising (CFIA 2011). CPCF labels should perform both these functions adequately and appropriately in order to protect and promote optimal infant and young child feeding (IYCF). There is however a lack of comprehensive formal guidance from international normative bodies on the appropriate marketing of CPCF, of which labelling practices are a sub‐set. The ‘International Code of Marketing of Breast‐milk Substitutes’ (the Code) (WHO 1981) offers little guidance in this regard as CPCF do not fall within its scope unless marketed or otherwise represented as a partial or total replacement of breastmilk. Other international guidelines, regulations and standards that offer limited guidance on the marketing of CPCF are highlighted in Box 1. Recognising the need for guidance, between 2007 and 2010, the Maternal, Infant and Young Child Nutrition Working Group (MIYCN WG) of the Ten Year Strategy to Reduce Vitamin and Mineral Deficiencies developed a working paper ‘Using the Code of Marketing of Breast‐milk Substitutes to Guide the Marketing of Complementary Foods to Protect Optimal Infant Feeding Practices’ (Quinn et al. 2010). This document describes how the marketing of CPCF (product labelling; advertising and promotion; marketing activities such as the sale, use and provision of information within the healthcare system) can be guided by the code and subsequent relevant World Health Assembly (WHA) resolutions in a manner that supports optimal IYCF. A study on the labelling practices of CPCF in South Africa, using the MIYCN WG interim guidance, concluded that CPCF labels do not sufficiently protect and promote optimal IYCF practices (Sweet 2012; Sweet et al. 2012). Examples of labelling practices that may interfere with optimal breastfeeding included: failure to recommend an age of introduction that is 6 months or more; labelling the product in a way that potentially promotes the manufacturer's infant or follow‐up formula (cross‐promotion); the use of phrases or images of infants that imply that the product is suitable for infants younger than 6 months; recommending a daily ration too large for a breastfed child. The gap in guidance led the Member States of the 65th WHA to request that the Director‐General ‘provide clarification and guidance on the inappropriate promotion of foods for infants and young children cited in resolution WHA63.23, taking into consideration the on‐going work of the Codex Alimentarius Commission’ (WHA 2012).

**Box 1. International guidelines, regulations and standards that offer guidance relevant to the marketing of commercially produced complementary foods**

**Table:** 

International Instruments	Scope of the instrument includes guidance relevant to the marketing, and practices related thereto of commercially produced complementary foods
The International code of marketing of breast milk substitutes (the Code) (WHO 1981)	Only when complementary foods are represented as suitable, with or without modification, for use as a partial or total replacement of breast milk.
Subsequent relevant WHA resolutions*	Offers limited guidance.
*Only the most relevant resolutions are listed.	• WHA Res. 63.23**:** Urges member states ‘to end inappropriate promotion of food for infants and young children and to ensure that nutrition and health claims shall not be permitted for foods for infants and young children, except where specifically provided for, in relevant Codex Alimentarius standards or national legislation’ (WHA 2010);
	• WHA Res. 49.15: Urges member states ‘to ensure that complementary foods are not marketed for or used in ways that undermine exclusive and sustained breast‐feeding’ (WHA 1996);
	• WHA Res. 39.28: Requests the director general to direct the attention of member states and other interested parties to the following: ‘any food or drink given before complementary feeding is nutritionally required may interfere with the initiation or maintenance of breastfeeding and therefore should neither be promoted nor encouraged for use by infants during this period’ (WHA 1986).
Codex Alimentarius Standards/Guidelines*	Offers limited guidance on product labelling.
*Only commodity standards and guidelines for complementary foods are listed, while relevant general Codex texts are not.	• Codex standard for canned baby foods (CODEX STAN 73–1981. Amendment 1983, 1985, 1987, 1989) (Codex Alimentarius 1981).
	• Codex standard for processed cereal‐based foods for infants and young children (CODEX STAN 74–1981, REV. 1–2006) (Codex Alimentarius 2006)
	• Guidelines on formulated complementary foods for older infants and young children (CAC/GL 8–1991) (Codex Alimentarius 1991 and revised in 2013)
The global strategy for infant and young child feeding (WHO, 2003)	Offers limited guidance.
	Appeals to all governments to protect, promote and support optimal infant and young child feeding, defined as exclusive breastfeeding for the first 6 months of life with continued breastfeeding up to 2 years or beyond; and to promote timely, adequate, safe and appropriate complementary feeding from 6 months of age.

Subsequently, the World Health Organization (WHO) formed a scientific and technical advisory group (STAG) that released a ‘Technical Paper on Definition of Inappropriate Promotion of Foods for Infants and Young Children’ in June 2013, ahead of 67th WHA in 2014 (WHO 2013). The 67th WHA acknowledged the work and requested that it be completed before the end of 2015 for consideration by Member States at the 69th WHA. To this end, the WHO STAG released a ‘Discussion paper: Clarification and Guidance on Inappropriate Promotion of Foods for Infants and Young Children’ in July 2015 for comment and consultation towards presentation at the WHA in May 2016 (WHO 2015).

At the national level, some countries have developed legislation that stipulates specific labelling requirements for foods for infants and young children that are wide ranging in the extent of the guidance. The Cambodian Sub‐Decree on Marketing of Products for Infant and Young Child Feeding (No. 133) (Kingdom of Cambodia 2005) contains provisions relevant to labelling practices for all products marketed as suitable from birth to 24 months of age, including that labels should have a statement on the superiority of exclusive breastfeeding for the first 6 months of life and sustained breastfeeding until 2 years and allows no images other than for illustrating preparation techniques. The Nepal Mother's Milk Substitutes (Control of Sale and Distribution) Act 2049 regulates products that are marketed as suitable from birth to 12 months (Government of Nepal 1992) and contains comprehensive labelling provisions requiring manufacturers to obtain approval for the labels of all relevant products from the Breastfeeding Protection and Promotion Committee and prohibiting the use of images on product labels other than to illustrate product preparation. Tanzania's National Regulations for Marketing of Breast‐milk Substitutes and Designated Products, first promulgated in 1994 and updated in 2013 (Ministry of Health & Social Welfare 2013), regulates products that are marketed as suitable from birth to 5 years of age and also prohibits images other than of product preparation and adds a further prohibition on any images of bottles and teats. Senegal enacted the Inter‐ministerial Decree Establishing the Conditions for Marketing Breast‐milk Substitutes in 1994 (Republic of Senegal 1994). This law is more limited than those of Nepal, Cambodia and Tanzania and focuses on controlling the promotion of breastmilk substitutes (BMS) in health facilities but provides very little guidance on labelling.

The aim of this study was to assess the labelling practices of CPCF available for sale in the most populous city/metropolitan area of Cambodia, Nepal, Senegal and Tanzania for compliance with international guidance including elements of the recommendations in the WHO STAG draft paper on ‘Definition of Inappropriate Promotion of Foods for Infants and Young Children’ (WHO 2013) and selected aspects of national legislation on the marketing of these foods.

## Methods

### Study design and research setting

In this cross‐sectional survey CPCF (as defined in Box 2), available for sale in the most populous city in Cambodia and Tanzania and the largest metropolitan areas in Nepal and Senegal, were purchased and the information provided on the labels assessed for adherence to international guidance and selected aspects of national legislation. Cambodia, Nepal, Senegal and Tanzania were selected as Helen Keller International (HKI) already had an active presence in these countries and they represented regional diversity. Based on research conducted by Sweet *et al*. (2012), where 81% of products found in three out of nine provinces in South Africa were available in the country's most populous province of Gauteng, it was expected that many of the products available nationally in the four study countries would be available in the largest city/metropolis. Data collection was conducted in Phnom Penh, Kathmandu Valley, Dakar Department and Dar es Salaam, representing 10% (NIS et al. 2011), 6% (MOHP et al. 2012), 21% (ANSD 2010) and 7% (NBS & ICF Macro 2011) of their countries' populations, respectively

**Box 2. Definition of CPCF used in this study**

Any commercially produced food or beverage product, excluding BMS (infant formula, follow‐up formula, growing‐up/toddler milks and formulas for special medical purposes) that contains a label indicating that the product is intended for children <2 years of age, by the following:

• Using the words baby/babe/infant/toddler/young child in the context of a child's age e.g. baby food (food for babies), not the size/maturity of the product e.g. baby potato (young potato),

• Recommending an age of introduction less than 2 years on the label, or

• Using an image of a child appearing younger than two years of age or an image/text of infant feeding (which could include a bottle).

Types of CPCF include, but are not limited to cereal/porridge, homogenised/pureed food, snacks/finger food, and tea/water/juice.

**Figure 1:**
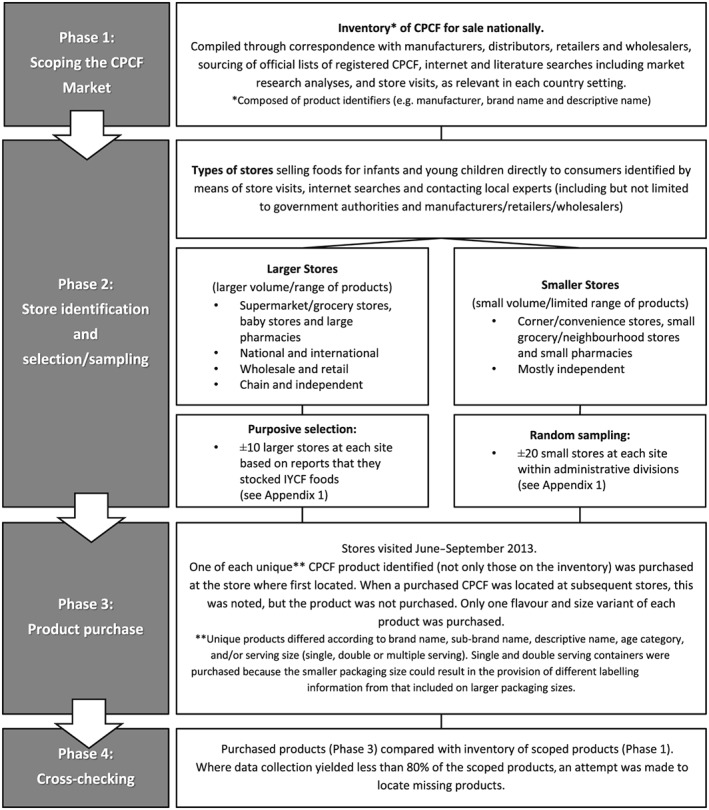
Data collection process for Commercially Produced Complementary Foods (CPCF).

Figure 1 presents a flow diagram of the data collection process. Phase 1, a scoping phase to create an inventory of CPCF available nationally, was designed to determine whether the product purchase conducted in each site (Phase 3) yielded at least 80% of the products theoretically available. A cross‐checking (Phase 4) was used to determine if the 80% had been reached or if additional product purchase was necessary. Phase 2 involved store identification and selection/sampling. It was anticipated that the majority of products manufactured by large/medium enterprises and sold nationally could be purchased from a purposive selection of larger store types (Sweet et al. 2012), while products manufactured by local small and medium enterprises, which might not be distributed through formal distribution channels, could be purchased from smaller stores. Smaller stores, of which there were many, were randomly sampled (see Appendix 1) as it was not possible to obtain a list of all smaller stores per study site.

### Data extraction, entry and analysis

Product labels were photographed or scanned and the images uploaded to a central digital folder. In each country, labels in the official national language [Khmer in Cambodia (Kingdom of Cambodia 2005); Nepali and/or English in Nepal (Government of Nepal 1970); French in Senegal (Republic of Senegal 1968); Swahili and/or English in Tanzania (Ministry of Health & Social Welfare 2006)] were professionally translated to English (unless accompanying English text was provided), and 10% were back translated to check for quality. Three labels containing Khmer text, seven labels with Nepali text, 63 labels with French text and one label with Swahili text required translation. If none of the label text was provided in the official language of the country, as was the case for 95.7% (*n* = 67) of labels from Phnom Penh and 8.3% (*n* = 7) of labels from Dakar Department, only the images on the label were assessed, as it was assumed that mothers/caregivers would only be literate in the country's official language, but would use pictures when making purchasing decisions. Thus, all labels from Kathmandu Valley and Dar es Salaam, 91.7% of labels from Dakar Department, and a mere 4.3% (*n* = 3) from Phnom Penh had their text and images assessed.

One researcher carried out data extraction by entering all predetermined categories of descriptive data from the product label into Microsoft Excel 2010 (Microsoft Corporation, Redmond, WA, USA). Label information from the data extraction database was used to complete a CPCF labelling practices checklist, a modified version of the checklist used by Sweet (2012) & Sweet et al. (2012) in South Africa. The Sweet *et al.* checklist was created using the guidance provided in the MIYCN WG working paper ‘Using the Code of Marketing of Breast‐milk Substitutes to Guide the Marketing of Complementary Foods to Protect Optimal Infant Feeding Practices’, which draws on guidance provided by the Code, subsequent relevant WHA resolutions, the WHO Global Strategy for IYCF and relevant Codex Alimentarius standards and guidelines. The checklist included elements that related to the five broad criteria in the draft WHO STAG document ‘Technical Paper on Definition of Inappropriate Promotion of Foods for Infants and Young Children’ (WHO 2013). Table 1 provides the checklist information pertaining to the results reported here.

**Table 1:** Commercially produced complementary food labelling practices checklist

Labelling practice question	Answers	Criteria for choosing answers	Reference for the question
**Age‐related recommendations and images**			
Does the product label specify a recommended age of introduction that is less than 6 months of age?	Yes	Recommended age of introduction is less than 6 months of age (180 days/the 7th‐month of life).	WHA resolution 39.28 (1986); WHA resolution 49.15 (1996); Global Strategy for IYCF (WHO 2003); Quinn, et al. 2010 (Section 3.1, p. 13–14; Section 4, p. 23).
	No*	Recommended age of introduction is 6 months of age (180 days/the 7th‐month of life) or later.	
	NA	The label does not specify an appropriate/recommended age of introduction.	
Does the product label include an appropriate/recommended age for use of the product that is 6 months (180 days) or more?	Yes*	Recommended age of introduction is from 6 months of age (180 days/the 7th‐month of life) or later.	WHA resolution 39.28 (1986); WHA resolution 49.15 (1996); Global Strategy for IYCF (WHO 2003); Quinn, et al. 2010. (Section 3.1, p. 13–14; Section 4, p. 23).
	No	Recommended age of introduction is before 6 months of age (180 days/the 7th‐month of life). Or no age of introduction is specified.	
Does the product label include images of babies appearing to be older than 6 months of age?	Yes*	Pictures of babies showing achievement of physical or developmental milestones clearly reached after 6 months of age: standing with assistance; hands‐and‐knees crawling; walking with assistance; standing alone; walking alone; one or more teeth; peddling a tricycle; running; holding objects such as a spoon/cup and self‐feeding; kicking a ball; standing on tip toes.	WHO MGRS 2006; Quinn, et al. 2010 (Section 3.1, p. 14–15; Section 4, p. 25).
		NB: If the label carries multiple images of children, all of the images have to qualify for a ‘Yes’ answer before the answer ‘Yes’ can be selected.	
	Unclear	Pictures of babies showing ‘Milestones: Other/Unclear’. NB: If the label carries multiple images of children, select unclear if none of the images qualify for a ‘No’ answer and at least one qualifies for an ‘unclear’ answer**.**	
	No	Pictures of infants/young children showing physical or developmental milestones commonly associated with infants 0 to 6 months of age such as holding a toy and shaking it; lying down; lying on stomach and pushing up to elbows; no teeth; reclining; sitting with support; sitting without support.	
		Or head shot of infant (including baby in mothers arms) with no physical or developmental milestones reached after 6 months displayed.	
		Or heavily stylized image of a baby with no physical or developmental milestones reached after 6 months displayed.	
		NB: If the label carries multiple images of babies, even if only one of the images qualifies for a ‘No’ answer, select ‘No’*.*	
	NA	No images of infants/young children on the label.	
**Infant and young child feeding messages**			
Does the product label include the following messages:			
The importance of exclusive breastfeeding for the first 6 months of life;	Yes*	A message including all three of the following concepts: exclusive; breastfeeding; and first 6 months.	Quinn, et al. 2010 (Section 3.1; p. 14–15; Section 4, p. 23).
	No	No message	
	Partial	A message including one or two of the three concepts: exclusive; breastfeeding; and first 6 months.	
The importance of the addition of complementary foods from 6 months of age with continued breastfeeding up to 2 years or beyond;	Yes*	A message including all three of the following concepts: the addition of complementary foods from 6 months; continued breastfeeding (after 6 months); up to 2 years or beyond.	Quinn, et al. 2010 (Section 3.1; p. 14–15; Section 4, p.23).
	Partial	A message including one or two of the three concepts.	
	No	No message	
**Cross‐promotion and invitations to interact on commercially produced complementary foods produced by breastmilk substitute manufacturers**			
In the case of manufacturers that produce both breastmilk substitutes and complementary foods, is the product labelled in a way that also promotes the company's infant or follow‐up formula by using similar:	Yes	Similarities in one or more of the listed elements.	Quinn, et al. 2010 (Section 3.2.2, p. 17; Section 4, p. 27).
	No*	None of the listed similarities.	
• colour schemes or designs; or	NA	Company does not sell infant formula/follow‐up formula/breastmilk substitutes in the country.	
• names; or			
• slogans, mascots or other symbols			
as used for their infant formula or follow‐up formula brands?			
In the case of manufacturers that produce both breastmilk substitutes and complementary foods, is the product labelled in a way that also promotes the company's breastmilk substitutes (e.g. infant or follow‐up formula) by including pack‐shots of such products on the label and/or directly referring to the company's Infant formula/follow‐up formula/growing up milk? (e.g. to prepare the cereal with the manufacturer's follow‐up formula)	Yes	Product contains front‐of‐pack shots of the manufacturers breastmilk substitute.	Code (WHO 1981) (Article 5.1).
		Product contains preparation instructions/infant feeding messages/claims that refer to the manufacturers breastmilk substitute (infant formula/follow up formula/growing up milk).	
	No*		
	NA	Company does not sell breastmilk substitutes (e.g. infant formula or follow‐up formula) in the country.	
In the case of manufacturers that produce both breastmilk substitutes and complementary foods, is there an invitation on the label to make contact (direct or indirect) with the company's marketing personnel?	Yes	E.g. ‘Contact our nutrition experts’ or a web link to a company sponsored baby club or IYCF information/ education service. Does not include the provision of company contact details for the purpose of reporting product defects or quality issues. Quick response code and website are always considered an invitation to contact; needs to be checked with other label content.	Code (WHO 1981) (Article 5.5); Quinn, et al. 2010 (Section 3.2.3, p. 17–18; Section 4, p. 27).
	No*	A customer care line, email address and postal address (without any other wording such as ‘contact our nutrition experts’) is considered to be company contact details for the purpose of reporting product defects or quality issues.
	NA	Company does not sell infant formula/follow‐up formula/breastmilk substitutes in the country.
**Serving size and daily ration**			
Does the product label include a proposed daily ration/serving (or recommended number of servings per day and serving)?	Yes*	Label provides both of the following: a proposed daily ration (even if calculated)/recommended number of servings per day and serving size.	Codex 1991; Quinn, et al. 2010 (Section 3.1, p. 15; Section 4, p. 24).
	Partial	Label provides one of the following: a proposed daily ration/recommended number of servings per day or serving size.	
	No	No proposed daily ration/recommended number of servings per day or serving size.	
Does the daily ration (or a recommended serving size combined with a recommended frequency of feeds per day) included on the product label exceed the recommended energy intake from complementary foods for a breastfed child provided below? For products where an age of introduction is not provided, answer the question for all age categories.			
6–8.9 months: 837 kJ/day (200 Kcal/day)	Yes	Greater than	PAHO/WHO 2003; Quinn, et al. 2010 (Section 3.1; p. 13–15; Section 4, p. 24).
	No*	Less than	
	Insufficient Information	No daily ration (nor a recommended serving size nor energy content) provided.	
	NA	Product not recommended for this age group (age of introduction from 9 months or older).	
9–11.9 months: 1255 kJ/day (300 Kcal/day)	Yes	Greater than	
	No*	Less than	
	Insufficient Information	No daily ration (nor a recommended serving size nor energy content) provided	
	NA	Product not recommended for this age group (age of introduction from 12 months or older).	
12–23.9 months: 2301 kJ/day (550 Kcal)	Yes	Greater than or equal to	
	No*	Less than	
	Insufficient information	No daily ration (nor a recommended serving size nor energy content) provided	
	NA	Product not recommended for this age group (age of introduction from 2 years or older).	

IYCF, infant and young child feeding; NA, not applicable.

* The answer reflecting ‘best practice’.

A second researcher randomly selected and cross‐checked 10% of the data. Disagreements regarding information extracted were resolved by consensus and where consensus could not be reached, and a third researcher made the final decision. Two researchers independently completed the checklist and reached consensus for all answers in Phnom Penh and Dar es Salaam. In 0.31% of checklist answers in Dakar Department and 0.65% in Kathmandu Valley, a third researcher made the final decision in consultation with the other researchers.

The data were imported into statistical software STATA v10 (StataCorp, College Station, TX, USA). Descriptive statistics were used to present a record of current labelling practices.

## Results

The number of CPCF products included in the sample was 70 in Phnom Penh, 22 in Kathmandu Valley, 84 in Dakar Department and 26 in Dar es Salaam. The most common product type was infant cereals in Kathmandu Valley and Dar es Salaam, and purees in Phnom Penh and Dakar Department (Table 2). Between 1.4% and 36.4% of products in all four sites were locally produced, and Nestlé, Danone and Heinz produced the largest number of products, except in Dar es Salaam where no Danone products were found (Table 3).

**Table 2:** Type of Commercially Produced Complementary Food products available for sale in Phnom Penh, Kathmandu Valley, Dakar Department and Dar es Salaam and manufacturers

	Phnom Penh *n* (%)	Kathmandu Valley *n* (%)	Dakar Department *n* (%)	Dar es Salaam *n* (%)
Type of product				
Infant cereals	17 (24.3)	21 (95.5)	36 (42.8)	20 (76.9)
Purees	30 (42.9)	1 (4.5)	39 (46.4)	4 (15.4)
Snack/finger foods	14 (20.0)	0	3 (3.5)	2 (7.7)
Juices/waters	9 (12.9)	0	6 (7.1)	0
Total products	70 (100.0)	22 (100.0)	84 (100.0)	26 (100.0)
Locally produced products	1 (1.4)	8 (36.4)	4 (4.8)	8 (30.8)
Imported products	69 (98.6)	14 (63.6)	80 (95.2)	18 (69.2)
Number of manufacturers	16	12	18	11
Manufacturers of products				
Nestlé	28* (40.0)	9 (40.9)	17 (20.2)	7 (26.9)
Danone	8† (11.4)	3‡ (13.6)	36† (42.9)	0
Heinz	11 (15.7)	1 (4.5%)	4 (4.8)	5 (19.2)
Cow & Gate	0	0	2 (2.4)	5 (19.2)
Other	23 (32.9)	9 (40.9)	25 (29.8)	9 (34.6)

* Includes both Nestlé and Gerber products as the latter is a subsidiary of the Nestlé Group.

† Includes both Danone and Bledina products as the latter is a company of the Danone Group.

‡ Includes Nutricia products and Farex brands as both are now part of the Danone Group.

**Table 3:** Commercially produced complementary food products for which the manufacturer also produces BMS and of those, labels using cross‐promotion, direct reference to the BMS and invitation to contact the manufacturer

	Phnom Penh *n* (%)	Kathmandu Valley *n* (%)	Dakar Department *n* (%)	Dar es Salaam *n* (%)
CPCF products for which the manufacturer also produced BMS:	24 (34.3)	12 (54.5)	59 (70.2)	12 (46.2)
Cross‐promotion	10 (41.2)	9 (75.0)	46 (78.0)	5 (41.7)
Direct reference to the BMS	2 (8.3)	0	9 (15.3)	0
Invitation to contact the manufacturer	18 (75.0)	0	54 (91.5)	10 (83.3)

CPCF, commercially produced complementary foods; BMS, breastmilk substitutes.

### CPCF Label characteristics

In all four sites, none of the CPCF labels were found to be compliant with all of the labelling checklist requirements.

### Age‐related recommendations and images of infants and young children

**Figure 2:**
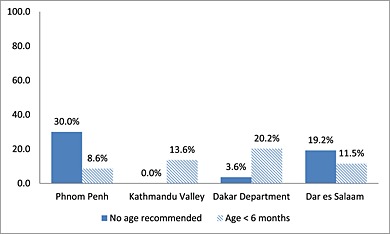
Percentage of commercially produced complementary food labels that provided no recommended age of introduction (months) or a recommended age less than 6 months.

Between 8.6% and 20.2% of products from all four sites recommended an age of introduction of <6 months. Most of these recommended use from 4 months except for two products from Kathmandu Valley which recommended use from 5 months. Kathmandu Valley was the only site where all products provided an age recommendation, while 3.6–30% of products in the remaining sites did not provide any age recommendation (Fig. 2).

**Figure 3:**
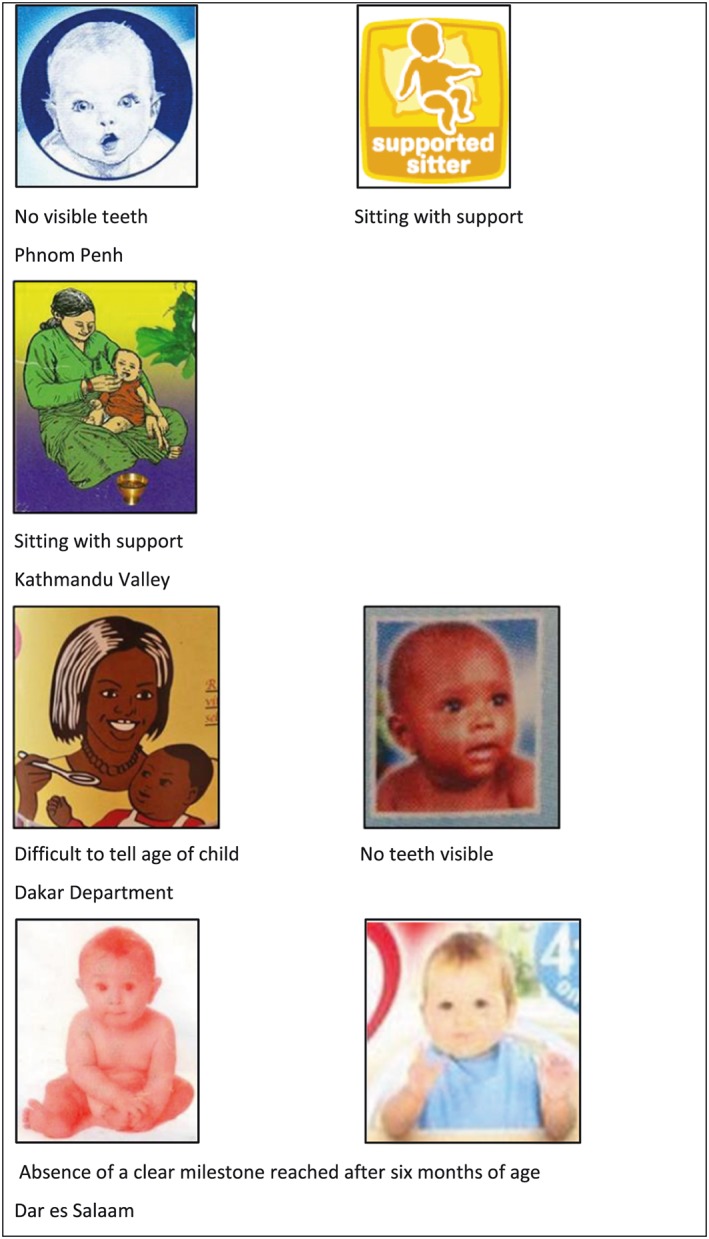
Images of infants displaying physical or developmental milestones commonly achieved before 6 months of age found on commercially produced complementary food labels in Phnom Penh, Kathmandu Valley, Dakar Department and Dar es Salaam.

Many labels included images of infants or young children: 37.1% in Phnom Penh, 22.7% in Kathmandu Valley, 14.3% in Dakar Department and 38.5% in Dar es Salaam, and some of these images suggested that they were suitable for infants <6 months of age (Fig. 3). An image of an infant displaying a developmental milestone commonly associated with infants <6 months of age, or no clear milestone reached after 6 months of age, was found on 24 CPCF labels in Phnom Penh, three labels in Kathmandu Valley, nine labels in Dakar Department and two labels in Dar es Salaam, representing 92.3%, 60%, 75% and 20% of the labels with images of infants/young children in each site, respectively.

### IYCF messages

A message stating the importance of exclusive breastfeeding for the first 6 months of life in the language required by national legislation was provided on 4.3% (*n* = 3), 27.3%, 3.6% (*n* = 3) and 26.9% of labels in Phnom Penh, Kathmandu Valley, Dakar Department and Dar es Salaam, respectively. Few labels (2.9% (*n* = 2) and 2.4% (*n* = 2)) in Phnom Penh and Dakar Department respectively and no labels in Kathmandu Valley and Dar es Salaam) provided a complete message including both the importance of the addition of complementary foods from 6 months of age and continued breastfeeding up to 2 years or beyond, in the language required by law. However, product labels more frequently provided one part of this message. In Kathmandu Valley, 63.6% recommended the addition of complementary foods from 6 months; 45.5% recommended continued breastfeeding but none recommended breastfeeding to 2 years or beyond. In Dakar Department 9.5% (*n* = 8) recommended introducing complementary foods at 6 months; 6.0% (*n* = 5) recommended continued breastfeeding; and 2.4% (*n* = 2) recommended continuing to breastfeed for 2 years or beyond. In Dar es Salaam, 19.2% (*n* = 5) recommended the addition of complementary foods from 6 months; 11.5% (*n* = 3) recommended continued breastfeeding and none recommended breastfeeding to 2 years or beyond.

The message ‘Breastfeeding is best for your baby’ was provided on all labels with Khmer text (4.3% of the total) in Phnom Penh, as well as 68.2%, 1.2% (*n* = 1) and 11.5% of labels in Kathmandu Valley, Dakar Department and Dar es Salaam, respectively. ‘Breastfeeding should continue as long as possible’ was stated on 27.3% of products in Kathmandu Valley. In Dakar Department, the most common message that was found on 4.7% (*n* = 4) of products was ‘Continue breastfeeding while introducing solids’. The most common message in Tanzania was ‘Exclusive breastfeeding is recommended for up to 6 months’ (26.9% of labels). Fifty‐five per cent of labels in Kathmandu Valley, 23.1% in Dar es Salaam, 2.4% in Dakar Department and 0% in Khmer in Phnom Penh used the term ‘weaning’.

### Cross‐promotion and invitations to interact on CPCF produced by BMS manufacturers

**Figure 4:**
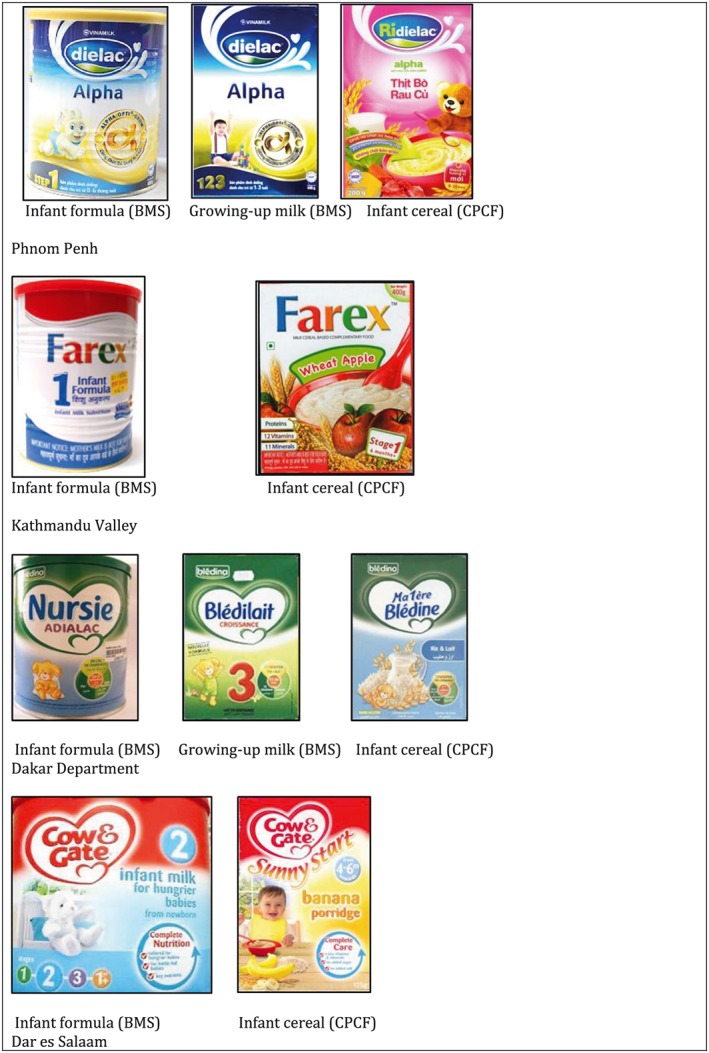
Examples of manufacturer's cross‐promotion (similar/same colour scheme, design and/or name) between commercially produced complementary foods (CPCF) and breastmilk substitute (BMS) products.

The percentage of CPCF products sold by manufacturers that also produce BMS ranged from 34.3–70.2% (Table 3). Of these, 41.2–78.0% products were labelled in a way that may be considered to promote the manufacturers' BMS products by using similar colour schemes, designs or names as used for their infant formula, follow‐up formula or growing‐up/toddler milk brands (examples provided in Fig. 4). In Phnom Penh and Dakar Department 8.2% and 15.3% of products, respectively, directly referred to the company's BMS by including a pack‐shot or recommending by name the use of the company's follow‐up formula. In addition, 75.0–91.5% of CPCF produced by BMS manufacturers included an invitation on the label, such as an invitation to join a baby club/to ‘contact our nutrition experts’, SMS line, website address or quick response codes, to make contact with the manufacturer.

### Daily ration and serving sizes

At each study site, while most labels provided a recommended serving size, few provided sufficient information to calculate a daily ration. For those labels that did provide sufficient information, the manufacturer's recommended serving size/daily ration was compared to the recommended daily energy intake from complementary foods for a breastfed child for three respective age categories, 6–8.9 months, 9–11.9 months and 12–23.9 months, based on the global daily energy needs from complementary foods and recommended number of meals for the breastfed child, given in Table 4 (PAHO/WHO 2003).

**Table 4:** The daily ration (or a recommended serving size combined with a recommended frequency of feeds per day) included on the product label that exceeded the recommended energy intake from complementary foods for a breastfed child*

Age group	Answer	Phnom Penh		Kathmandu Valley		Dakar Department		Dar es Salaam	
		*n* (%)	Total *n* for age group	*n* (%)	Total *n* for age group	*n* (%)	Total *n* for age group	*n* (%)	Total *n* for age group
6–8.9 months: 837 kJ/day (200 Kcal/day)	Exceeded recommendation	9 (14.5)	62	10 (55.6)	18	17 (23.3)	73	10 (40.0)	25
	Did not exceed recommendation	3 (4.8)		0		3 (4.1)		1 (4.0)	
	Insufficient information	50 (80.6)		8 (44.4)		53 (72.6)		14 (56.0)	
9–11.9 months: 1255 kJ/day (300 Kcal/day)	Exceeded recommendation	4 (6.2)	65	13 (61.9)	21	8 (10.3)	78	2 (8.0)	25
	Did not exceed recommendation	4 (6.2)		0		2 (2.6)		7 (28.0)	
	Insufficient information	57 (87.6)		8 (38.1)		68 (87.1)		16 (64.0)	
12–23.9 months: 2301 kJ/day (550 Kcal)	Exceeded recommendation	5 (7.1)	70	4 (18.1)	22	2 (2.4)	83	0	26
	Did not exceed recommendation	3 (4.3)		10 (45.5)		7 (8.4)		8 (30.8)	
	Insufficient information	62 (88.6)		8 (36.4)		74 (89.2)		18 (69.2)	

* Includes product labels with daily ration or serving size plus number of meals (meal frequency) used to calculate daily ration.

As shown in Table 4, in Phnom Penh 14.5% (*n* = 9), 6.2% (*n* = 4) and 7.1% (*n* = 5) of applicable products exceeded the recommended daily energy intake for the three age categories, respectively. In Kathmandu Valley, 55.6% (*n* = 10), 62% (*n* = 13) and 18.1% (*n* = 4) of applicable products exceeded the recommended daily energy intake for the three age categories. In Dakar Department, 23.3% (*n* = 17), 10.3% (*n* = 7) and 2.4% (*n* = 2) of applicable products exceeded the recommended daily energy intake for the three age categories, respectively. In Dar es Salaam, 40% (*n* = 10), 8% (*n* = 2) and none of applicable products exceeded the recommended daily energy intake for the three age categories, respectively. In all four countries for all three age groups, there was often insufficient information provided to calculate the daily ration.

## Discussion

This study assessed the labels of CPCF sold in Phnom Penh, Kathmandu Valley, Dakar Department and Dar es Salaam and the findings reveal many and significant areas where they are non‐compliant with national legislation, the spirit of the Code, subsequent relevant WHA resolutions and recommendations made in the document ‘Using the Code of Marketing of Breast milk Substitutes to Guide the Marketing of Complementary Foods to Protect Optimal Infant feeding Practices’ (Quinn *et al.* 2010). The labelling practices of manufacturers assessed in this study show that they do no support optimal IYCF practices and best‐practice.

The Global Strategy for IYCF emphasises that appropriate evidence‐based feeding practices are essential for attaining and maintaining proper nutrition and health and that inadequate knowledge about appropriate foods and feeding practices are often a greater determinant of malnutrition than lack of food. The strategy recognises the role of CPCF for some mothers who have the means to buy them and the knowledge and facilities to prepare and feed them safely (WHO 2003). The labels of CPCF have a role to play in supporting optimal IYCF.

We found that the majority of CPCF in the sample were imported, and although 11–18 CPCF manufacturers were recorded per site, 54.0–77.0% of CPCF were manufactured by four multinational companies. These markets are thus dominated by imported products from a few manufacturers and, contrary to what is encouraged by the Global Strategy for IYCF (WHO 2003), there are limited locally produced products.

### Age‐related recommendations and images of infants and young children

International guidance recommends exclusive breastfeeding for the first 6 months of life and the introduction of safe, appropriate complementary foods from 6 months of age together with continued breastfeeding to 2 years of age and beyond (PAHO/WHO 2003; WHO 2003), and labels should support this by providing an appropriate age of introduction. It is concerning that 13.6–38.6% of CPCF labels in the four sites failed to provide an appropriate age recommendation in text, by either not providing an age recommendation or by recommending an age of introduction of less than 6 months. A possible explanation for the labels promoting the early use of CPCF is that almost half (49%) of all products from all four sites were imported from Europe, where the European Food Safety Authority (EFSA) declared that ‘the introduction of complementary food into the diet of healthy term infants in the EU between the age of four and six months is safe and does not pose a risk for adverse health effects’ (EFSA 2009). This divergence of opinions (WHO versus EFSA) and regulatory requirements (EU countries versus low and middle income countries) poses a challenge in a world where global trade is increasing, and national law enforcement in many low and middle income countries is weak. This finding highlights the need for greater global harmonization of regulations pertaining to foods for infants and young children. As a demonstration of their support of optimal IYCF at the global level manufacturers, especially those exporting products to a number of countries, should label all CPCF with an age of introduction from 6 months or later (Quinn et al. 2010; WHO 2013).

Furthermore, product labels may encourage the early introduction of CPCF by using images of infants showing physical or developmental milestones commonly associated with infants younger than 6 months of age and as a result it was suggested that if images of infants are permitted by the country, only images of infants older than 6 months of age and showing achievement of a physical or developmental milestone clearly reached after 6 months should be used on CPCF (Quinn et al. 2010; WHO, 2015). This study shows that there is a wide range between the study sites (20.0–92.3%) of labels with images of infants/young children that fail to use an appropriate image.

In Nepal, Cambodia and Tanzania, images of infants/young children, regardless of their age, are a violation of the law, which only permits images illustrating preparation methods on foods for infants up to 12 months of age in Nepal, children up to 2 years in Cambodia and children up to 5 years in Tanzania (Government of Nepal 1992; Kingdom of Cambodia 2005; Ministry of Health and Social Welfare 2013). As a number of CPCF in these three countries violated national law in this regard, there is a need for manufacturers to ensure that they comply with the regulations in countries where their products are available for sale and for countries to strengthen enforcement of existing regulations.

### IYCF messages

Labels can inform on IYCF practices; educational messages should support optimal IYCF – defined as ‘exclusive breastfeeding from birth for the first six months of life (180 days) and starting from six months of age, feeding safe and appropriate complementary foods, along with continued breastfeeding for up to two years of age or beyond’ (WHO 2003). In this study, only a small percentage (3.6–27.3%) of labels were found to provide accurate and complete messages encouraging exclusive breastfeeding and almost none (0.0–2.9%) provided accurate and complete messages regarding the appropriate introduction of complementary foods together with continued breastfeeding. ‘Breastfeeding should continue as long as possible’, found on over a quarter of CPCF in Kathmandu Valley and Dar es Salaam, is incomplete and thus misleading and may undermine maternal confidence in the ability to continue breastfeeding, previously shown to be significantly related to breastfeeding duration and level (Blyth et al. 2002).

As BMS directly competes with breastmilk in the infant's diet, the Code requires BMS labels to carry a ‘statement of the superiority of breastfeeding’ (WHO 1981). It appears that in an effort to apply the principles of the Code to the labelling of CPCF, manufacturers of more than two‐thirds of CPCF in Kathmandu Valley included this, or a similar message, ‘Mother's milk is best for your baby’. As infants from 6  months of age require complementary foods in addition to breastmilk, this message is insufficient if used alone on complementary food labels as it implies that breastmilk is all that is required and is preferable to complementary foods. Such messaging may have negative consequences in a country such as Nepal where the late introduction of complementary foods is a concern (MOHP et al. 2012). Also, more than half of CPCF labels in Kathmandu Valley used the term ‘weaning’, which can be interpreted as ‘the complete cessation of breastfeeding’ (Canadian Paediatric Society 2004) and its use can infer that the product is suitable as a replacement for breastmilk.

### Cross‐promotion and invitations to interact on CPCF produced by BMS manufacturers

Breastmilk substitutes, both under the Code and legislation in Cambodia (unless given specific Ministry of Health approval), Nepal, and Tanzania may not be promoted directly to the consumer, yet 41.2–78.0% of CPCF sold by companies that also sell BMS were labelled in a way that potentially also promoted the company's formula products by using the same or similar brand names or logos, colour schemes or designs, slogans, mascots or symbols. Following the voluntary adoption of the Code in Australia and consequent restrictions on the promotion of infant formula, manufacturers have increased their advertising of follow‐up formula, growing‐up milk and CPCF and brand‐focused promotion to consumers (Smith and Blake 2013). Cross‐promotion (or ‘brand cross‐over promotion’, ‘brand‐stretching’ or ‘line extension’ (Berry et al. 2011)) uses one product to advertise another (Cambridge Dictionaries Online 2014). When presented with a product, which is an extension of a known brand, the manufacturer's shared use of the brand's attributes (colour, name, design, and so on) through the product range has been shown to result in the consumer taking into account what they know about the one product, and applying that knowledge to the new product under the same brand, thus building brand loyalty (Park et al. 1991). Advertising of follow‐up formula, growing‐up milk and CPCF products, which share brand attributes with infant formula, could result in the *de‐facto* promotion of the company's infant formula and thus circumvent the Code and national regulations (Berry et al. 2010; Berry et al. 2012). Therefore, Australian researchers recommend passing legislation that prohibits the advertising of all products sharing a brand identity with infant formula (Berry et al. 2010), a view shared by others (Quinn, et al. 2010; WHO 2013).

A related practice, in the form of invitations to interact from manufacturers of CPCF who also sell BMS, was also noted. This is often an invitation to join a baby club/to ‘contact our nutrition experts’/website address/quick response codes, which is both a violation of the Code, which states in Article 5.5 that BMS manufacturers' marketing personnel should not seek indirect contact of any kind with pregnant women or with mothers of infants and young children, and contrary to global guidance (WHO 1981; Quinn et al. 2010).

### Daily ration and serving sizes

Two key challenges to achieving optimal IYCF during the complementary feeding phase are ensuring adequate nutrient content of foods and maintaining breastfeeding up to the age of 24 months and beyond. CPCF labels should recommend daily rations and serving sizes that provide energy within the daily requirements for breastfed children because excessive consumption could result in the displacement of continued breastfeeding as well as other locally available and appropriate foods (WHO 2015; Quinn et al. 2010). Few CPCF products provided a daily ration (and of those that did, many exceeded the daily energy recommendation for complementary foods for a breastfed child). Of concern was that just over a third (36%) of labels that recommended a single serving size (for 6–8.9 month‐olds) exceeded the PAHO/WHO guidelines (data not shown). Furthermore, over half of CPCF cereal labels that included a daily ration exceeded the recommended daily energy intake for complementary foods for a breastfed child (data not shown), raising concerns about portion sizes recommended by CPCF manufacturers, especially in the light of the burgeoning double burden of under‐ and over‐nutrition in low and middle income countries (Ng et al. 2014).

### Limitations

Although efforts were made to include as many available CPCF products per country as possible, there may be products available outside of the study sites that were not included. In an attempt to reduce interpretation bias of the images appearing on the labels, two researchers independently completed the checklist and where discrepancies were found and consensus was not possible, a third researcher made the final decision. Product labelling serves as a potential marketing tool for food manufacturers and is thus currently under the spotlight as countries look to address the inappropriate promotion of foods for infants and young children. However, labelling is only one element of the marketing mix and, as such, is not fully indicative of the extent of marketing or its influence on IYCF practices.

This study only included CPCF products purchased in the selected stores, hence the results may not be generalised to represent other channels via which CPCF products reach mothers/caregivers, such as internet purchasing or home sales.

## Conclusions

Labelling practices of CPCF included in this study do not fully comply with international guidance on the promotion of CPCF and selected aspects of national legislation, and so do not sufficiently protect and promote optimal IYCF practices. Inappropriate practices that were particularly prevalent included the lack of appropriate age of introduction, lack of accurate and complete IYCF messages, recommended portion sizes and daily rations in excess of the daily requirements for breastfed children, and cross promotion between CPCF and BMS produced by the same manufacturer. Such practices have the potential to undermine public health messages regarding optimal breastfeeding and the timely and appropriate introduction of complementary foods and encourage the displacement of continued breastfeeding and other locally available and appropriate foods. In addition, many of the images depicting infants and young children appearing on the labels were considered to be potentially misleading. Corporates that manufacture any food or beverage for infants and young children can – and do – influence their feeding, and therefore need to take greater responsibility to protect and promote optimal IYCF practices.

### Recommendations

There is a need for clarification and detailed guidance from normative bodies on a wide range of practices pertaining to the labelling and inappropriate promotion of CPCF, to assist governments in setting relevant regulations and prohibiting manufacturers of CPCF and BMS from undertaking any form of cross‐promotion between these products. The work of the WHO STAG, in providing clarification and guidance, will be valuable (WHO 2015). All manufacturers, especially multinational companies, manufacturing CPCF products must take greater responsibility for monitoring the labelling, distribution and sale (directly or indirectly) of their products, to ensure that they do not violate the national regulations in the country of sale. This is especially important when products are imported from one country to another with stricter regulations. In addition, manufacturers should comply with global normative guidance as a minimum standard when national regulations are absent. Review and revision of legislation regulating the labelling of CPCF in Cambodia, Nepal, Senegal and Tanzania, to ensure it is specific and comprehensive, is necessary. Equally important is the need for improved monitoring and enforcement of regulations in the four countries. Further research is required regarding a number of label elements including IYCF messages and images carried on foods for IYCF, to determine understanding and the extent to which they influence, either positively or negatively, a mother/caregivers choice of foods available to feed her child.

## Funding source

Funding was received from the Bill & Melinda Gates Foundation (BMGF). The authors affirm their independence from the funder. The funder played no part in the study design, collection, analysis or interpretation of data, or in the writing of the report, or in the decision to submit the article for publication.

## Conflicts of interest

Jane Badham and Rosalyn Ford have acted as nutrition consultants to the food industry but have not worked for manufacturers of breastmilk substitutes or complementary foods.

## Contributions

LS, JB and EZ conceptualised and LS and CP designed the study. KM, NYSG, ANC, IA and CM assisted in developing the national level sampling methodology and collected the data, supported by LS and CP. CP and RF entered, and CP, RF and AF analysed and interpreted the data with assistance from LS and JB. LS, CP, RF, AF and JB drafted the paper. EZ critically reviewed the paper.

## APPENDIX 1 Store sampling methodology by study site

**Table:**

**Study site**	**Administrative divisions (AD)**		**No. of AU randomly sampled (% of total)**	**No. small stores visited per randomly sampled AU** (corner/convenience; small grocery/neighborhood; independent pharmacy)	**No. large stores purposively sampled per study site** (supermarket/grocery stores, baby stores; pharmacies)	**Total no. stores sampled**
	**1st level**	**No. of administrative units (AU) per 1st level AD**				
**Senegal: Dakar Department (DD)**	Borough:	Borough communes:		1 corner store and 1 neighborhood store per borough commune; 1 independent pharmacy for every 2 borough communes (max. 4 )		
	1. Almadies	4	2			
	2. Dakar‐Plateau	4^a^	2			
	3. Grand‐Dakar	6	3			
	4. Parcelles Assainies	4	2			
	**Total:**	**18**	**9 (50%)**	**22 (9;9;4)**	**9 (6;0;3)**	**31**
**Nepal: Kathmandu Valley (KV)**	Urban municipality^b^:	Wards:		2 stores per ward: 1 independent pharmacy for every 2 wards (max. 5);1–2 corner stores per ward		
	1. Kathmandu Metropolitan City	35	7			
	2. Lalitpur Sub‐metropolitan City	22	4			
	**Total:**	**57**	**11 (19%)**	**22 (17;0;5)**	**9 (9;0;0)**	**31**
**Cambodia: Phenom Phen (PP)**	Khan:	Urban sangkat:		1 small grocery store and 1 convenience store per sangkat; 1 independent pharmacy for every 2 sangkat		
	1. Chamkar Mon	12	2			
	2. Doun Penh	11	2			
	3. Prampir Meakkakra	8	2			
	4. Tuol Kouk	10	2			
	**Total:**	**41**	**8 (20%)**	**18** c **(7;7;4)**	**11 (6;4;1)**	**29**
**Tanzania: Dar es Salam (DeS)**	Municipal council:	Urban wards:		2 stores per ward: 1–2 convenience stores per ward; 1–2 independent pharmacies per municipal council		
	1. Ilala	7	2			
	2. Kinondoni	19	5			
	3. Temeke	11	3			
	**Total:**	**37**	**10 (27%)**	**20 (16;0;4)**	**10 (8;0;2)**	**30**
	**Grand Total:**			**82**	**39**	**121**

a Gorée is not included as it is an island and only borough communes that form part of the mainland are included in this study.

b Of the 5 municipal areas in KV, Bhakptapur municipality, Kirtipur municipality, and Madhyapur Thimi municipality were excluded from the study due to challenges experienced with access to stores. Although these areas are officially classified as urban, some areas are rural or very sparsely populated as well as the areas having poor road infrastructure, making it difficult to implement the study methodology with regard to store sampling.

c Of the original 20 small stores, two were excluded because it was determined during data extraction that the products purchased at these stores did not meet the inclusion criteria for the study.
